# Comparative investigation of interactions of hydrogen, halogen and tetrel bond donors with electron-rich and electron-deficient π-systems†

**DOI:** 10.1039/c9ra08007d

**Published:** 2019-10-15

**Authors:** Mahmoud A. A. Ibrahim, Ossama A. M. Ahmed, Nayra A. M. Moussa, Sabry El-Taher, Hussien Moustafa

**Affiliations:** Chemistry Department, Faculty of Science, Minia University Minia 61519 Egypt m.ibrahim@compchem.net; Department of Chemistry, Faculty of Science, Cairo University Giza Egypt

## Abstract

Recently, noncovalent interactions in complexes and crystals have attracted considerable interest. The current study was thus designed to gain a better understanding of three seminal types of noncovalent interactions, namely: hydrogen, halogen and tetrel interactions with π-systems. This study was performed on three models of Lewis acids: X_3_–C–H, F_3_–C–X and F–T–F_3_ (where X = F, Cl, Br and I; and T = C, Si, Ge and Sn) and three π-systems as Lewis bases: benzene (BZN), 1,3,5-trifluorobenzene (TFB) and hexafluorobenzene (HFB). Quantum mechanical calculations, including geometrical optimization, molecular electrostatic potential (MEP), maximum positive electrostatic potential (*V*_s,max_), Point-of-Charge (PoC), potential energy surface (PES), quantum theory of atoms in molecules (QTAIM) and noncovalent interaction (NCI) calculations, were carried out at the MP2/aug cc-pVDZ level of theory. The binding energies were additionally benchmarked at the CCSD(T)/CBS level. The results showed that: (i) the binding energies of the X_3_–C–H⋯π-system complexes were unexpectedly inversely correlated with the *V*_s,max_ values on the hydrogen atom but directly correlated with the X atomic sizes; (ii) the binding energies for the F_3_–C–X⋯π-system and F–T–F_3_⋯π-system complexes were correlated with the σ-hole magnitudes of the X and T atoms, respectively; and (iii) for the F_3_–C–F⋯π-system complexes, the binding energy was as strong as the π-system was electron-deficient, indicating the dominating nucleophilic character of the fluorine atom. NCI analysis showed that the unexpected trend of X_3_–C–H⋯π-system binding energies could be attributed to additional attractive interactions between the X atoms in the X_3_–C–H molecule and the carbon atoms of the π-system. Furthermore, the I_3_–Sn–H molecule was employed as a case study of hydrogen, halogen and tetrel interactions with π-systems. It was found that hydrogen and halogen interactions of the I_3_–Sn–H molecule correlated with the electron-richness of the π-system. In contrast, tetrel interactions correlated with the electron deficiency of the π-system.

## Abstract 2

Three seminal types of noncovalent interaction, namely: hydrogen, halogen and tetrel interactions with π-systems, were investigated using quantum mechanical calculations.

## Graphical abstract

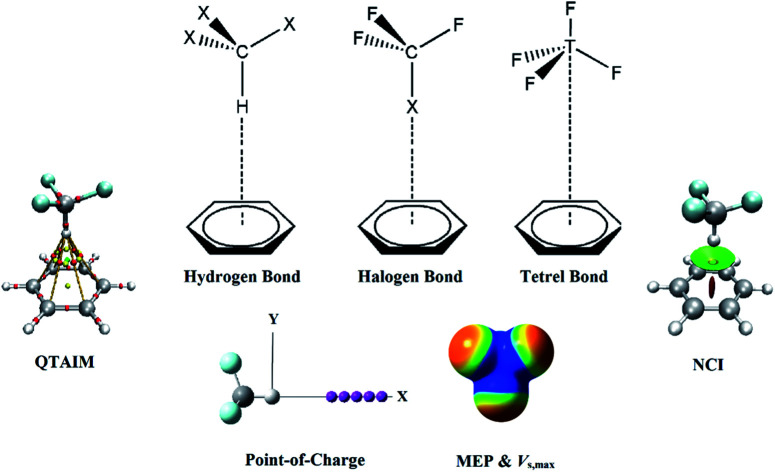

## 1. Introduction

**Fig. 1:**
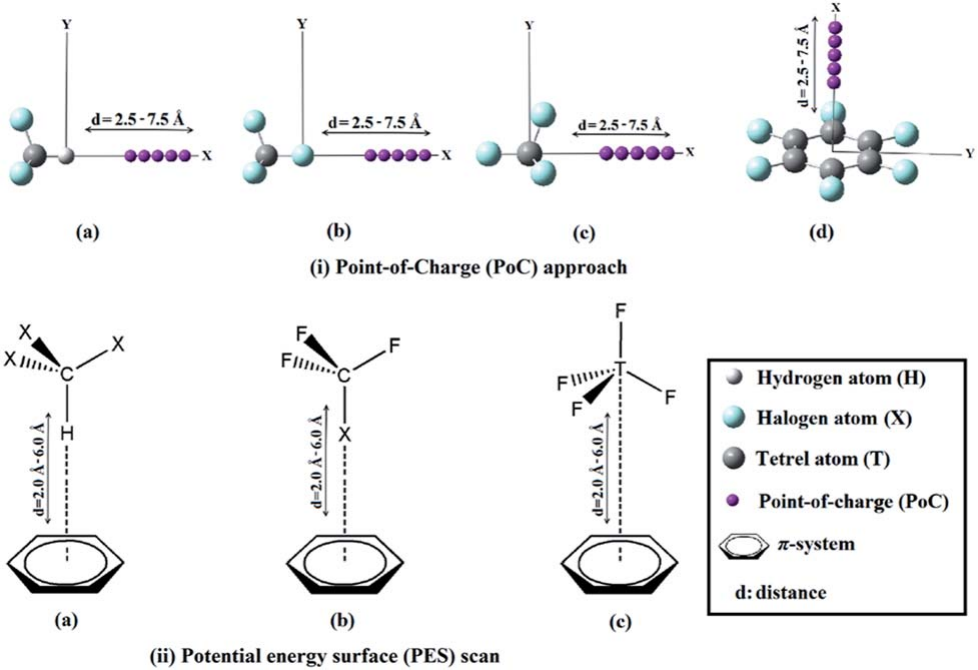
Schematic representation for (i) the Point-of-Charge (PoC) calculations for (a) X_3_–C–H, (b) F_3_–C–X, (c) F–T–F_3_, and (d) π-system, where X = F, Cl, Br and I, T = C, Si, Ge and Sn, and π-system = BNZ, TFB and HFB, and (ii) potential energy surface (PES) scans for (a) X_3_–C–H⋯π-system, (b) F_3_–C–X⋯π-system and (c) F–T–F_3_⋯π-system complexes.

Noncovalent interactions play crucial roles in multidisciplinary fields including crystal engineering^1,2^ and drug discovery.^3–5^ Probably, the most prominent type of noncovalent interaction is the hydrogen bond which plays a vital role in a plethora of chemical and biochemical processes.^6–10^ Along with the hydrogen bond, the σ-hole interaction is another remarkable type of noncovalent interaction.^11^ The occurrence of the latter interaction is mainly attributed to the existence of an electron-deficient region (called a σ-hole^12^) on the molecular electrostatic potential surface along the extension of the covalently bonded Group IV–VII elements in the periodic table. Group IV–VII elements are referred to as σ-atoms and have the potential to interact through their σ-holes with Lewis bases to form tetrel,^13–15^ pnicogen,^16–18^ chalcogen^19–21^ and halogen^22–24^ bonds, respectively. Both the size and magnitude of the σ-hole correlate with the atomic size of the σ-atom with all other parameters held fixed.^25^ Thus, the fluorine atom has the smallest σ-hole in size and magnitude, among all halogens. Equivalently, the carbon atom has the smallest σ-hole of all tetrel atoms. While hydrogen, halogen and tetrel bond donors are capable of acting as Lewis acids, Lewis base candidates can be anions, lone-pair donors or π-systems.^25–28^ A careful literature search revealed that σ-hole⋯π-system interactions have not been yet sufficiently, let alone systematically, studied. Hence, a comparative investigation is required to assess the relative strengths of hydrogen, halogen and tetrel bond interactions with a series of π-systems. This study intended to contribute to the fulfilment of this purpose. In this work, hydrogen, halogen and tetrel bond donors will be studied primarily as Lewis acids that interact with electron-rich and electron-deficient π-systems. For the studied monomers, geometrical optimization, molecular electrostatic potential (MEP) and maximum positive electrostatic potential (*V*_s,max_) calculations will be performed. Moreover, Point-of-Charge (PoC) approach will be implemented to investigate the extent to which a molecule is stabilized or destabilized by approaching negative and positive charges (*i.e.* Lewis bases and Lewis acids, respectively).^29–33^ Hydrogen, halogen and tetrel interactions will be then studied in X_3_–C–H⋯, F_3_–C–X⋯ and F–T–F_3_⋯π-system complexes, respectively. For the studied complexes, potential energy surface (PES) scans will be performed in specific orientations to give the investigated interactions (see Fig. 1). The binding energies of the complexes will be also benchmarked at CCSD(T)/CBS level of theory. Quantum theory of atoms in molecules (QTAIM) and the noncovalent interaction (NCI) index calculations will be utilized to investigate the nature of the interactions under study. Finally, noncovalent interactions in I_3_–Sn–H⋯π-system complex will be investigated and factors empowering the interactions will be highlighted. The findings of this research afford profound insights into π-system-based noncovalent interactions that are essential to many chemical and biochemical processes.

## 2. Computational methodology

In the current study, X_3_–C–H, F_3_–C–X and F–T–F_3_ models (where, X= F, Cl, Br and I; T = C, Si, Ge and Sn) were employed as Lewis acid centres to form hydrogen, halogen and tetrel bonds, respectively with π-systems including benzene (BZN), 1,3,5-trifluorobenzene (TFB) and hexafluorobenzene (HFB). All the monomers were firstly optimized at MP2/aug-cc-pVDZ level of theory,^34,35^ with treating Br, I, Ge and Sn atoms with aug-cc-pVDZ-PP basis set.^36^ The molecular electrostatic potentials (MEPs) were generated for all the studied monomers and mapped on 0.002 au electron density contours. The maximum positive electrostatic potential (*V*_s,max_) values were computed using Multiwfn 3.5 software.^37^ To inspect the potentiality of the studied monomers to participate in electrostatic interactions with Lewis bases and acids, the Point-of-Charge (PoC) approach was implemented.^29–33^ In this approach, negatively and positively charged points with values of ±0.50 au were utilized to simulate the effect of Lewis bases and Lewis acids, respectively. In the PoC approach, H/σ-atom⋯/π-system⋯PoC distance was taken to be in the range 2.5 Å to 7.5 Å with a step size of 0.1 Å (see Fig. 1). The molecular stabilization energy (*E*_stabilization_) was estimated at MP2/aug-cc-pVDZ (with PP functions for Br, I, Ge and Sn atoms) level of theory and calculated as follows:

1 *E*_stabilization_ = *E*_molecule⋯PoC_ − *E*_molecule_

For X_3_–C–H⋯, F_3_–C–X⋯ and F–T–F_3_⋯π-system complexes, the optimized monomers were positioned in a specific orientation to give the desired interactions as shown in Fig. 1. For complexes, potential energy surface (PES) scans were performed in H/σ-atom⋯π-system bond in the range of 2.0 Å to 6.0 Å far from the π-system centroid and with a step size of 0.1 Å (see Fig. 1). The binding energies were estimated at MP2/aug-cc-pVDZ (with PP functions for Br, I, Ge and Sn) level of theory and the basis set superposition error (BSSE) was eliminated *via* the counterpoise correction method.^38^ The binding energies of the studied complexes were also computed at CCSD(T)/CBS level of theory according to the following equations:^39^

2 *E*_CCSD(T)/CBS_ = Δ*E*_MP2/CBS_ + Δ*E*_CCSD(T)_

where:

3 Δ*E*_MP2/CBS_ = (64*E*_MP2/aug-cc-pVQZ_ − 27*E*_MP2/aug-cc-pVTZ_)/37

4 Δ*E*_CCSD(T)_ = *E*_CCSD(T)/aug-cc-pVDZ_ − *E*_MP2/aug-cc-pVDZ_

Furthermore, the nature of noncovalent interactions in the studied complexes was investigated in terms of the electron density and its derivatives using the quantum theory of atoms in molecules (QTAIM).^40^ Bond critical points (BCPs) and bond paths were extracted and depicted. Topological parameters including electron density, Laplacian and total electron energy density were calculated. Noncovalent interaction (NCI) indices^41^ were also computed and NCI plots for the studied complexes were generated. The colouring scale of *ρ* was from −0.035 to 0.020 au. The QTAIM and NCI calculations were performed at MP2/aug-cc-pVDZ level of theory (with PP functions for Br, I, Ge and Sn). Finally, the interplay of hydrogen, halogen and tetrel bonds in I_3_–Sn–H⋯π-system complex as case study was investigated. All the quantum mechanical calculations were carried out using Gaussian 09 software;^42^ while QTAIM and NCI analyses were performed using Multiwfn 3.5 software.^37^ QTAIM and NCI diagrams were visualized using Visual Molecular Dynamics (VMD) software.^43^

## 3. Results & discussion

### 3.1 MEP, *V*_s,max_ and PoC

**Fig. 2:**
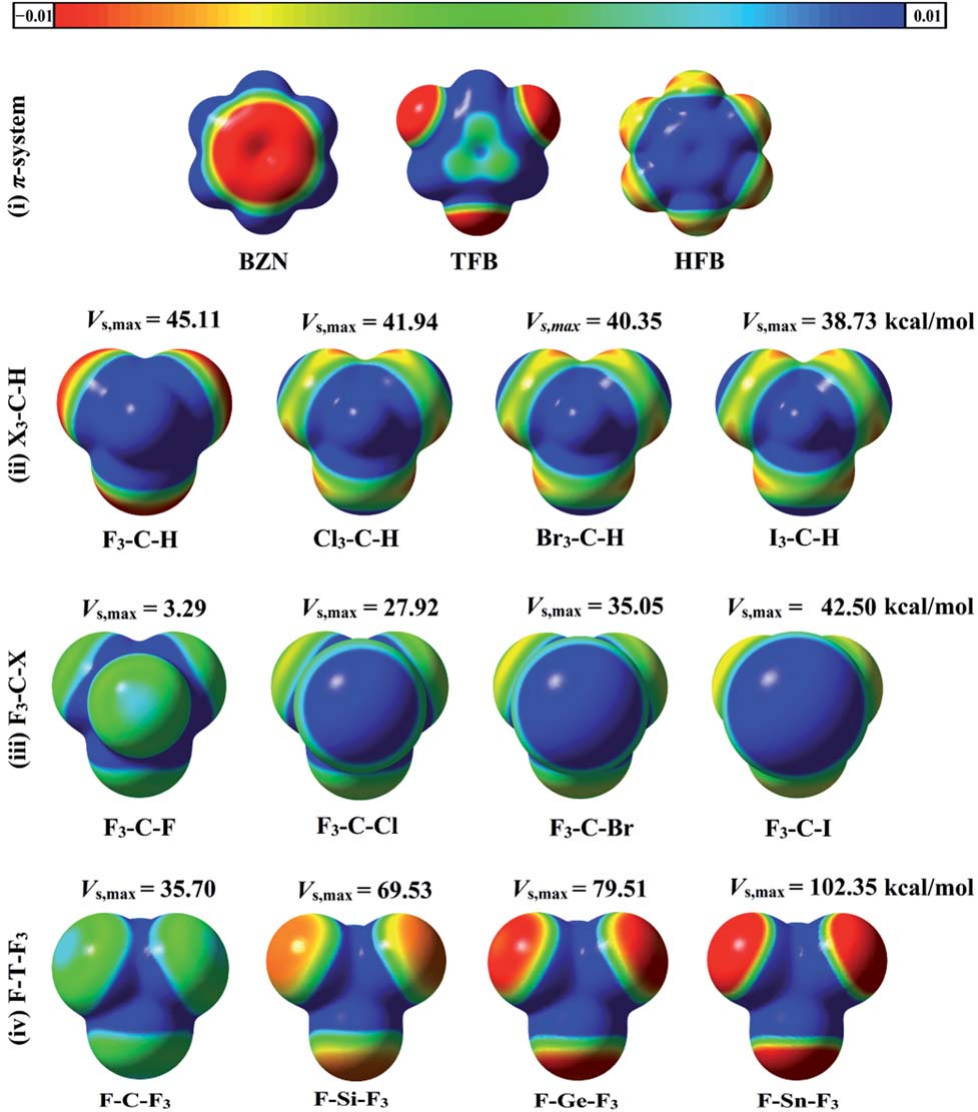
Molecular electrostatic potential (MEP) maps of (i) π-system (BNZ, TFB, and HFB), (ii) X_3_–C–H, (iii) F_3_–C–X, and (iv) F–T–F_3_, where X = F, Cl, Br and I, T = C, Si, Ge and Sn. The colour scale varies from −0.01 (red) to +0.01 (blue) au. The maximum positive electrostatic potentials (*V*_s,max_) values of hydrogen, halogen, and tetrel atoms in the studied molecules are in kcal mol^−1^.

Molecular electrostatic potentials (MEPs) are powerful for predicting electrophilic and nucleophilic sites on molecular surfaces.^44^ MEPs for the optimized hydrogen, halogen and tetrel bond donors were generated and mapped on 0.002 au electron density contour (see computational methodology section for details). To compute the magnitude of molecular electrostatic potentials, *V*_s,max_ calculations were carried out. MEP maps and *V*_s,max_ values for all studied monomers are depicted in Fig. 2.

As seen in Fig. 2, the MEP maps and *V*_s,max_ values of hydrogen atoms in X_3_–C–H molecules increased as the electron withdrawing power of the attached X atoms increased in the order I_3_–C–H < Br_3_–C–H < Cl_3_–C–H < F_3_–C–H. Moreover, the sizes and magnitudes of σ-holes for the F_3_–C–X and F–T–F_3_ molecules were found to be directly correlated with the atomic sizes of the σ-atoms (*i.e.*, halogen and tetrel atoms). For the studied π-systems, the electrostatic potentials above the benzene carbon ring were negative and became more positive with increasing number of fluorine substituents in the order BZN < TFB < HFB.

**Fig. 3:**
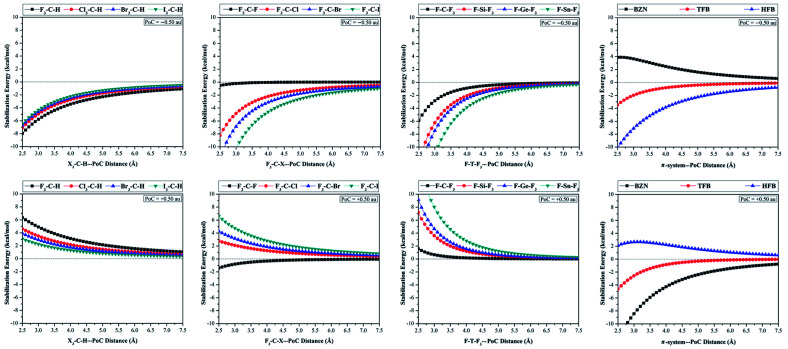
Molecular stabilization energies of X_3_–C–H⋯, F_3_–C–X⋯, F–T–F_3_⋯ and π-system⋯PoC systems (where X = F, Cl, Br and I, T = C, Si, Ge and Sn, and π-system = BNZ, TFB, and HFB) in the presence of ± 0.50 au PoC at H/σ-atom⋯/π-system⋯PoC distances from 2.5 to 7.5 Å.

With the Point-of-Charge (PoC) approach, the molecular stabilization energies of the studied monomers towards the approaching charges were assessed and compared. In this approach, the H/σ-atom⋯/π-system⋯PoC distances were taken in the range 2.5 Å to 7.5 Å with a step size of 0.1 Å (see computational methodology section for details). Molecular stabilization energy curves were generated for all studied monomers and are depicted in Fig. 3. The molecular energies calculated at H/σ-atom⋯/π-system⋯PoC distance of 2.5 Å are summarized in Table 1.

**Table 1:** Molecular stabilization energies (*E*_stabilization_, in kcal mol^−1^) of the X_3_–C–H, F_3_–C–X, F–T–F_3_, and π-system (where X = F, Cl, Br and I, T = C, Si, Ge and Sn, and π-system = BNZ, TFB, and HFB) at H/σ-atom⋯/π-system⋯PoC distance of 2.5 Å in presence of ± 0.50 au PoCs

H bond donor	*E*_stab._	X bond donor	*E*_stab._	T bond donor	*E*_stab._	π system	*E*_stab._
**PoC value = −0.50 au**							
F_3_–C–H	−7.97	F_3_–C–F	−0.53	F–C–F_3_	−5.88	BZN	3.87
Cl_3_–C–H	−7.09	F_3_–C–Cl	−8.24	F–Si–F_3_	−12.31	TFB	−3.48
Br_3_–C–H	−6.74	F_3_–C–Br	−11.70	F–Ge–F_3_	−14.52	HFB	−10.1
I_3_–C–H	−6.47	F_3_–C–I	−17.54	F–Sn–F_3_	−21.06		

**PoC value = +0.50 au**							
F_3_–C–H	6.36	F_3_–C–F	−1.41	F–C–F_3_	1.58	BZN	−12.29
Cl_3_–C–H	4.62	F_3_–C–Cl	2.78	F–Si–F_3_	7.20	TFB	−4.65
Br_3_–C–H	3.91	F_3_–C–Br	4.29	F–Ge–F_3_	9.19	HFB	2.12
I_3_–C–H	3.06	F_3_–C–I	6.64	F–Sn–F_3_	15.19		

As seen in Fig. 3, X_3_–C–H⋯PoC systems showed an expected electrophilic character of hydrogen atom with significant stabilization energies in the presence of negative PoC and destabilization energies with positive PoC. For negative PoC, molecular stabilization energies decreased (*i.e.*, less negative) in the order F_3_–C–H > Cl_3_–C–H > Br_3_–C–H > I_3_–C–H. This trend was expected as *V*_s,max_ was largest in F_3_–C–H and lowest in I_3_–C–H (see Fig. 2). When positive PoC was incorporated, molecular destabilization energies were observed to decrease (*i.e.*, less positive) in the same order of F_3_–C–H > Cl_3_–C–H > Br_3_–C–H > I_3_–C–H. For F_3_–C–X and F–T–F_3_ molecules, molecular stabilization energies were observed in the presence of negative PoC and increased as the σ-hole size of X and T atoms increased. For positive PoC, molecular destabilization energies were observed for all the investigated molecules except F_3_–C–F. From Table 1, molecular stabilization energies of F_3_–C–F⋯PoC at 2.5 Å were observed with values of −0.53 and −1.41 kcal mol^−1^ in the presence of PoC of −0.50 and +0.50 au, respectively. This unexpected molecular stabilization energy for F_3_–C–F in presence of ± 0.50 au PoCs may be attributed to very weak electrophilic character and relatively higher nucleophilic character of the fluorine atom.

From the molecular stabilization energy curves, the π-systems under study exhibited diverse attitudes towards the incorporated PoCs. According to Fig. 3, the nucleophilic character of BZN was apparent in the destabilization and stabilization energy of BZN in the presence of negative and positive PoCs, respectively. For instance, the molecular energies for BZN were 3.87 and −12.29 kcal mol^−1^ at 2.5 Å with −0.50 and +0.50 au PoCs, respectively.

Contrary to BZN, HFB showed an electrophilic character of the π-system. At HFB⋯PoC distance of 2.5 Å, molecular energies were −10.10 and 2.12 kcal mol^−1^ in the presence of −0.50 and +0.50 au PoCs, respectively.

Interestingly, TFB revealed both electrophilic and nucleophilic characters with stabilization energies in case of both negative and positive PoCs. For instance, the molecular stabilization energies at TFB⋯PoC distance of 2.5 Å were found to be −3.48 and −4.65 kcal mol^−1^ in the presence of −0.50 and +0.50 au PoCs, respectively. This might be explained by the inductive polarization effect of negative/positive PoC (*i.e.* Lewis base/acid) on the π-system (*i.e.* TFB).^33,45–47^

### 3.2 Potential energy surface (PES) scan

**Fig. 4:**
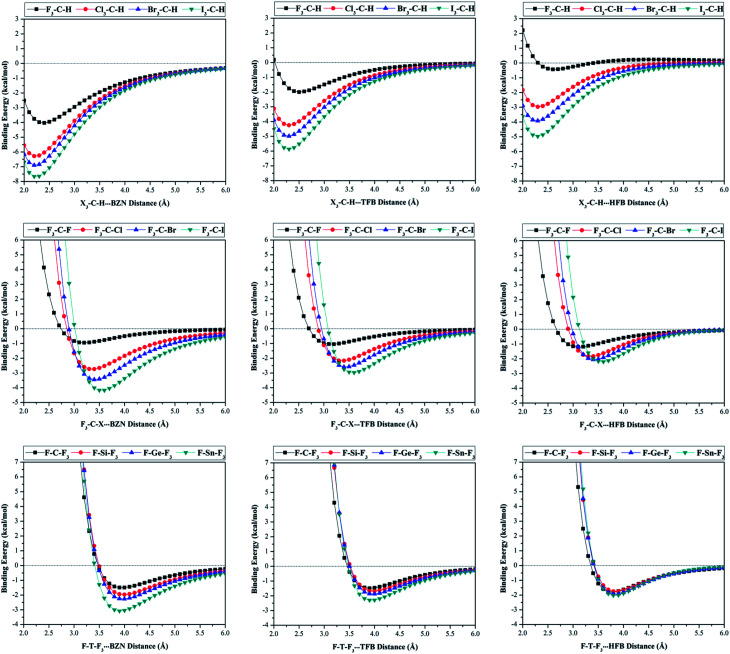
Binding energies calculated at MP2/aug-cc-pVDZ(PP) level of theory for X_3_–C–H⋯, F_3_–C–X⋯ and F–T–F_3_⋯π-system complexes (where X = F, Cl, Br and I, T = C, Si, Ge and Sn, and π-system = BNZ, TFB, and HFB) at H/σ-atom⋯π-system distances from 2.0 to 6.0 Å with a step size of 0.1 Å.

For the purpose of the study, potential energy surface (PES) scans were carried out for X_3_–C–H⋯, F_3_–C–X⋯ and F–T–F_3_⋯π-system complexes at distances from 2.0 Å to 6.0 Å (see computational methodology section for details). The generated PESs are depicted in Fig. 4. Binding energies for the studied complexes at the most favourable H/σ-atom⋯π-system distance were also calculated at CCSD(T)/CBS level of theory and summarized in Table 2.

**Table 2:** Binding energies calculated at MP2/aug-cc-pVDZ and CCSD(T)/CBS levels of theory for X_3_–C–H⋯, F_3_–C–X⋯, and F–T–F_3_⋯π-system complexes at the most favourable H/σ-atom⋯π-system distance

Noncovalent interaction	π-system	Bond donors	Distance^a^ (Å)	*E*_MP2/aug-cc-pVDZ_ b (kcal mol^−1^)	*E*_CCSD(T)/CBS_ (kcal mol^−1^)
Hydrogen-bond	BZN	F_3_–C–H	2.39	−4.03	−4.28
		Cl_3_–C–H	2.23	−6.29	−5.87
		Br_3_–C–H	2.22	−6.91	−6.39
		I_3_–C–H	2.24	−7.69	−7.18
	TFB	F_3_–C–H	2.51	−2.00	−2.08
		Cl_3_–C–H	2.30	−4.25	−3.80
		Br_3_–C–H	2.28	−5.00	−4.46
		I_3_–C–H	2.29	−5.88	−5.41
	HFB	F_3_–C–H	2.63	−0.43	−0.35
		Cl_3_–C–H	2.31	−2.97	−2.46
		Br_3_–C–H	2.28	−3.93	−3.35
		I_3_–C–H	2.29	−4.98	−4.53
Halogen-bond	BZN	F_3_–C–F	3.19	−0.95	−0.97
		F_3_–C–Cl	3.35	−2.75	−2.61
		F_3_–C–Br	3.41	−3.45	−3.27
		F_3_–C–I	3.55	−4.19	−4.19
	TFB	F_3_–C–F	3.15	−1.05	−1.11
		F_3_–C–Cl	3.36	−2.18	−1.95
		F_3_–C–Br	3.43	−2.59	−2.37
		F_3_–C–I	3.57	−2.98	−2.93
	HFB	F_3_–C–F	3.11	−1.20	−1.30
		F_3_–C–Cl	3.34	−1.85	−1.55
		F_3_–C–Br	3.42	−2.06	−1.78
		F_3_–C–I	3.57	−2.20	−2.11
Tetrel-bond	BZN	F–C–F_3_	3.95	−1.50	−1.60
		F–Si–F_3_	3.99	−1.97	−2.28
		F–Ge–F_3_	3.97	−2.26	−2.69
		F–Sn–F_3_	3.91	−3.08	−4.03
	TFB	F–C–F_3_	3.91	−1.49	−1.59
		F–Si–F_3_	3.97	−1.68	−1.92
		F–Ge–F_3_	3.96	−1.88	−2.24
		F–Sn–F_3_	3.94	−2.30	−2.95
	HFB	F–C–F_3_	3.78	−1.79	−1.91
		F–Si–F_3_	3.84	−1.76	−1.93
		F–Ge–F_3_	3.83	−1.93	−2.21
		F–Sn–F_3_	3.83	−2.02	−2.61

a The most favourable at H/σ-atom⋯π-system distance based on the depicted curves in Fig. 4.

b PP functions were implemented for Br, I, Ge and Sn atoms.

From data presented in Fig. 4, it is generally noticeable that all investigated complexes had significant negative binding energies. This reveals that hydrogen, halogen and tetrel bond donors have capability to favourably interact with both electron-rich and electron-deficient π-systems.

Contrary to expectations, the binding energies of X_3_–C–H⋯π-system complexes increased (*i.e.*, more negative) with increasing X atomic size in order F_3_–C–H < Cl_3_–C–H < Br_3_–C–H < I_3_–C–H. For instance, the binding energies of F_3_–C–H⋯, Cl_3_–C–H⋯, Br_3_–C–H⋯ and I_3_–C–H⋯BZN complexes were found to be −4.28, −5.87, −6.39 and −7.18 kcal mol^−1^, respectively.

Interestingly, this is inversely correlated to *V*_s,max_ values of the hydrogen atoms in X_3_–C–H molecules. The same trend was also observed for X_3_–C–H⋯TFB and ⋯HFB complexes. This indicates that X_3_–C–H⋯π-system binding energy is ruled by other noncovalent interactions rather than C–H⋯π-system interactions. Therefore, further investigation on the nature of X_3_–C–H⋯π-system interaction is required (see NCI analysis section).

A comparison of the X_3_–C–H⋯BZN, ⋯TFB and ⋯HFB complexes revealed that binding energy decreased as positivity of the electrostatic potential of π-system increased. For instance, the binding energies for the Br_3_–C–H⋯π-system complexes were found to be −6.39, −4.46 and −3.35 kcal mol^−1^ for Br_3_–C–H⋯BZN, ⋯TFB and ⋯HFB, respectively. This binding energy pattern is in agreement with previous results.^27,48^

For the halogen bond donors, binding energies of the F_3_–C–X⋯π-system complexes increased as the σ-hole size on halogen atom increased (*i.e.*, atomic size). For instance, the following binding energy trend was observed in F_3_–C–X⋯BZN complexes: F_3_–C–I⋯BZN > F_3_–C–Br⋯BZN > F_3_–C–Cl⋯BZN > F_3_–C–F⋯BZN with binding energies of −4.19, −3.27, −2.61 and −0.97 kcal mol^−1^, respectively. Moreover, binding energy decreased as positivity of the electrostatic potential of π-system increased. For instance, binding energy was found to decrease according to the order F_3_–C–Br⋯BZN > F_3_–C–Br⋯TFB > F_3_–C–Br⋯HFB with values of −3.27, −2.37 and −1.78 kcal mol^−1^, respectively. This trend may be understood in light of the nature of the interaction between the positive σ-hole and the negative sites of the π-system. For the F_3_–C–F⋯π-system, the binding energy trend was found to be reversed in the order F_3_–C–F⋯HFB > F_3_–C–F⋯TFB > F_3_–C–F⋯BZN with relatively low binding energies of −1.30, −1.11 and −0.97 kcal mol^−1^, respectively. This unexpected trend is consistent with PoCs results which may be explained in terms of the nucleophilic character of the fluorine atom being dominant over its electrophilic nature (*i.e.* fluorine atom prefers to act as a Lewis base rather than as a Lewis acid).

Similar to halogen bond donors, the F–T–F_3_⋯π-system binding energies were found to increase with increasing atomic size of the tetrel atom (*i.e.* σ-hole size). For instance, binding energies in F–T–F_3_⋯BZN complexes were found to be −1.60, −2.28, −2.69 and −4.03 kcal mol^−1^ for T = C, Si, Ge and Sn, respectively. The trend of F–T–F_3_⋯π-system interactions through BZN, TFB and HFB with same F–T–F_3_ molecule was rather irregular (see Table 2). This may be indicative of the role of F_3_ atoms from the F–T–F_3_ molecule in F–T–F_3_⋯π-system interactions.^29^ In the next section, a well-informed insight into the F–T–F_3_⋯π-system interactions will be gained through noncovalent interaction (NCI) index.

### 3.3 QTAIM analysis

**Fig. 5:**
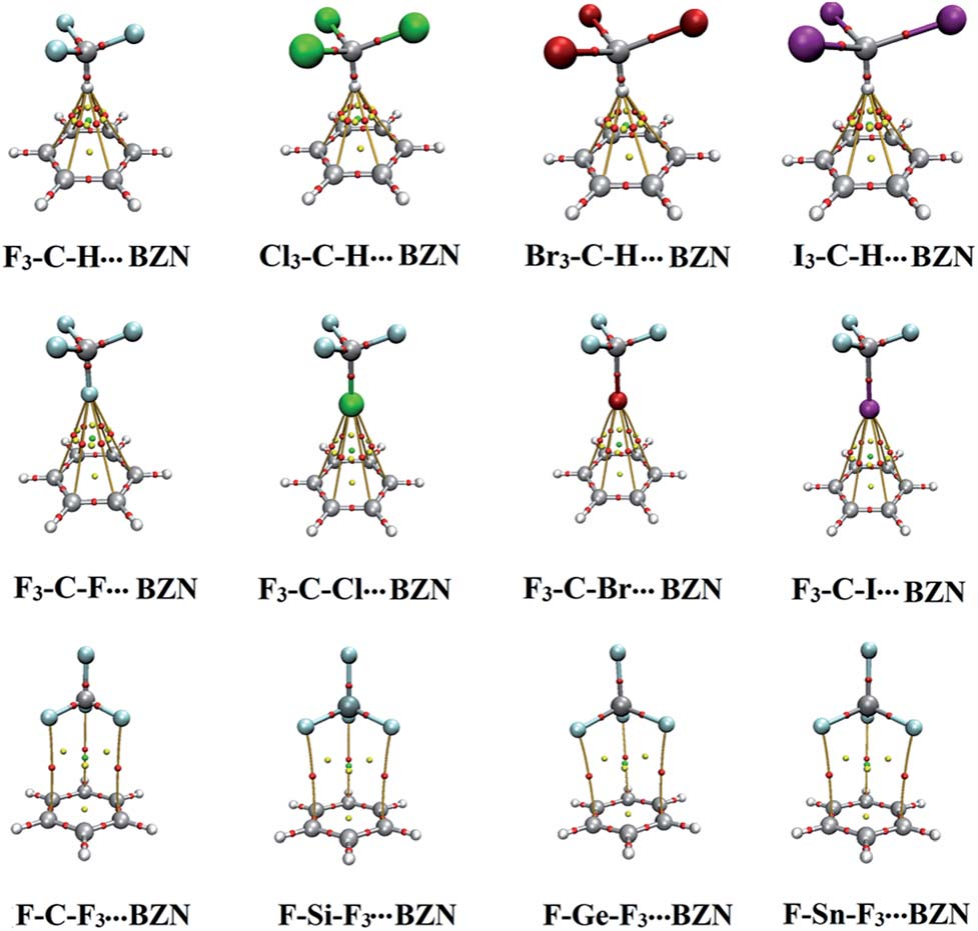
QTAIM diagrams of X_3_–C–H⋯, F_3_–C–X⋯ and F–T–F_3_⋯BNZ complexes (where X = F, Cl, Br and I, and T = C, Si, Ge and Sn). Red dots indicate the locations of bond critical points on bond paths between the monomers at the most favourable H/σ-atom⋯BNZ distance.

The quantum theory of atoms in molecules (QTAIM) is very informative on the nature of noncovalent bonding.^40,49–51^ In this study, QTAIM analysis was performed for X_3_–C–H⋯, F_3_–C–X⋯ and F–T–F_3_⋯π-system complexes at the most favourable H/σ-atom⋯π-system distances and the corresponding BCPs and bond paths were generated and visualized (Fig. S1†). BCPs and bond paths of X_3_–C–H/F_3_–C–X/F–T–F_3_⋯BZN complexes, as an example, are shown in Fig. 5. BCP characteristics, including the total energy density (H_b_), the Laplacian of the electron density (∇^2^*ρ*_b_) and electron density (*ρ*_b_), were also computed and tabulated in Table 3.

**Table 3:** Topological parameters including total energy density (H_b_, au), Laplacian (∇^2^*ρ*_b_, au), and electron density (*ρ*, au) at bond critical points (BCPs) calculated for the X_3_–C–H⋯, F_3_–C–X⋯, and F–T–F_3_⋯π-system complexes at the most favorable H/σ-atom⋯π-system distance

Noncovalent interaction	π-system	Bond donors	H_b_ (au)	∇^2^*ρ*_b_ (au)	*ρ*_b_ (au)
Hydrogen-bond	BZN	F_3_–C–H	0.00073	0.02420	0.00729
		Cl_3_–C–H	0.00076	0.03085	0.00955
		Br_3_–C–H	0.00079	0.03145	0.00971
		I_3_–C–H	0.00080	0.03065	0.00947
	TFB	F_3_–C–H	0.00067	0.02085	0.00618
		Cl_3_–C–H	0.00072	0.02855	0.00882
		Br_3_–C–H	0.00075	0.02960	0.00912
		I_3_–C–H	0.00076	0.01225	0.01144
	HFB	F_3_–C–H	0.00058	0.01770	0.00519
		Cl_3_–C–H	0.00067	0.02855	0.00891
		Br_3_–C–H	0.00070	0.03005	0.00936
		I_3_–C–H	0.00072	0.02980	0.00926
Halogen-bond	BZN	F_3_–C–F	0.00066	0.01370	0.00312
		F_3_–C–Cl	0.00050	0.01430	0.00464
		F_3_–C–Br	0.00054	0.01480	0.00497
		F_3_–C–I	0.00043	0.01380	0.00521
	TFB	F_3_–C–F	0.00068	0.01500	0.00345
		F_3_–C–Cl	0.00047	0.01440	0.00470
		F_3_–C–Br	0.00049	0.01460	0.00497
		F_3_–C–I	0.00039	0.01360	0.00522
	HFB	F_3_–C–F	0.00068	0.01630	0.00378
		F_3_–C–Cl	0.00045	0.01530	0.00495
		F_3_–C–Br	0.00046	0.01520	0.00515
		F_3_–C–I	0.00035	0.01390	0.00533
Tetrel-bond	BZN	F–C–F_3_	0.00063	0.01200	0.00313
		F–Si–F_3_	0.00068	0.01320	0.00352
		F–Ge–F_3_	0.00071	0.01430	0.00393
		F–Sn–F_3_	0.00079	0.01740	0.00493
	TFB	F–C–F_3_	0.00065	0.01300	0.00345
		F–Si–F_3_	0.00069	0.01380	0.00373
		F–Ge–F_3_	0.00071	0.01480	0.00408
		F–Sn–F_3_	0.00076	0.01670	0.00476
	HFB	F–C–F_3_	0.00074	0.01600	0.00422
		F–Si–F_3_	0.00082	0.01700	0.00447
		F–Ge–F_3_	0.00086	0.01820	0.00485
		F–Sn–F_3_	0.00092	0.01980	0.00536

According to data presented in Fig. S1,† noncovalent BCPs and bond paths were observed in all the studied complexes. The numbers of BCPs and bond paths were dependent on the nature of studied complex. For X_3_–C–H⋯π-system complexes, six BCPs between the hydrogen atom and the six carbon atoms of the π-system were identified whereas for tetrel bond-containing complexes three BCPs between the three coplanar fluorine atoms in F–T–F_3_ and the three carbon atoms in the π-system were observed. This indicates the intriguing role of F_3_ atoms in the F_3_–T–F⋯π-system interaction that was reported in our previous work.^29^ For halogen bond-containing complexes, six BCPs and three BCPs were identified in F_3_–C–X⋯BZN/HFB and TFB complexes, respectively.

For all the studied complexes, H_b_ at the BCP had positive values from 0.00035 au (in F_3_–C–I⋯HFB) to 0.00092 au (in F–Sn–F_3_⋯HFB) indicating the closed-shell nature of X_3_–C–H⋯π-system interactions. Generally, there was a correlation between H_b_ values and corresponding binding energies. For instance, H_b_ values for X_3_–C–H⋯TFB complexes for X = F, Cl, Br and I were observed to be 0.00067, 0.00072, 0.00075 and 0.00076 au with binding energies of −2.08, −3.80, −4.46 and −5.41 kcal mol^−1^, respectively.

The closed-shell nature of interaction was also pronounced in the relatively low values of *ρ*_b_ and the positivity of ∇^2^*ρ*_b_ indicating electronic charge depletions along the bond path (see Table 3). Generally, values of *ρ*_b_ were observed to increase as binding energies increased. For instance, *ρ*_b_ values in F_3_–C–X⋯TFB complexes were 0.00345, 0.00470, 0.00497 and 0.00522 au with binding energies of −1.11, −1.95, −2.37 and −2.93 kcal mol^−1^ for X = F, Cl, Br and I, respectively.

### 3.4 NCI analysis

**Fig. 6:**
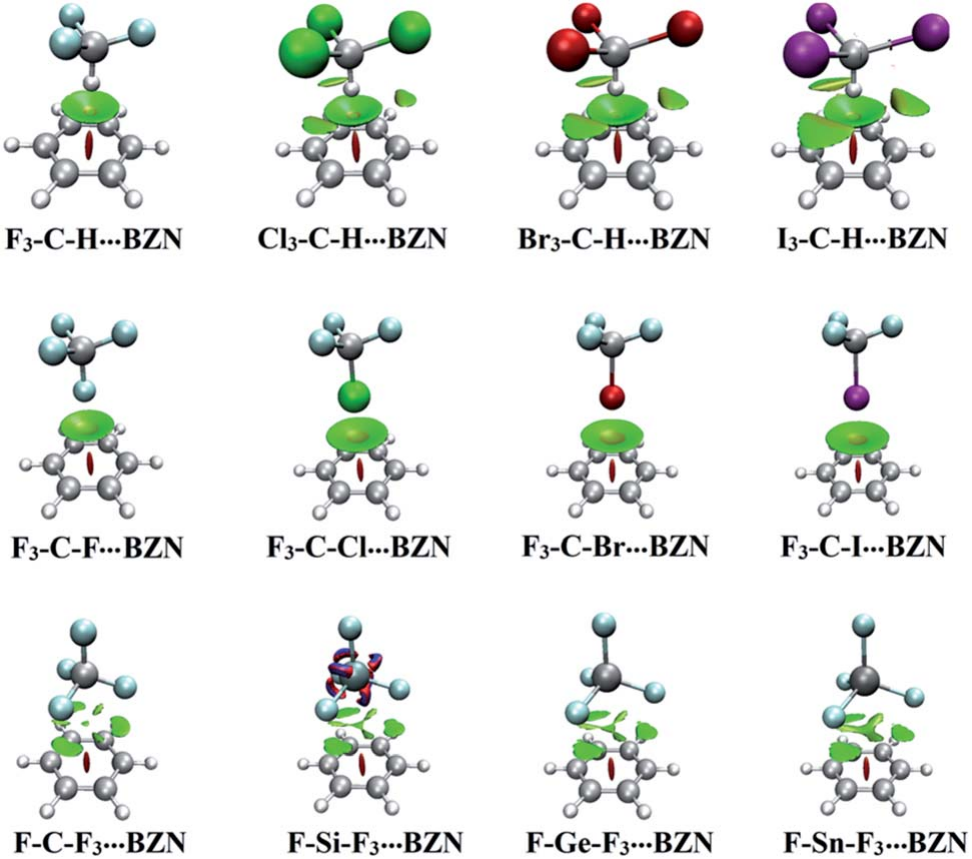
Noncovalent interaction (NCI) diagrams of X_3_–C–H⋯, F_3_–C–X⋯ and F–T–F_3_⋯BNZ complexes (where X = F, Cl, Br and I, and T = C, Si, Ge and Sn). The isosurfaces are plotted with a reduced density gradient value of 0.50 au and colored from blue to red according to sign(*λ*_2_)*ρ* ranging from −0.035 (blue) to 0.020 (red) au.

Noncovalent interaction (NCI) index relies fundamentally on reduced density gradient (RDG) to inspect regions of noncovalent bonding.^41^ NCI index is simpler and less restrictive than the rigorous QTAIM theory but occasionally gives indications of long range interactions that cannot be predicted by QTAIM theory.^52^ For the systems under study, RDG isosurfaces of 0.50 au value were generated and depicted in Fig. S2.†Fig. 6 shows the NCI diagrams of X_3_–C–H/F_3_–C–X/F–T–F_3_⋯BZN complexes. The colour scale of sign(*λ*_2_)*ρ* was from −0.035 (blue) to 0.020 (red), where *λ*_2_ is the second eigenvalue of the Hessian matrix and *ρ* is the electron density.

As seen in Fig. 6, NCI analysis revealed the occurrence of noncovalent interactions between hydrogen, halogen and tetrel bond donors with electron-rich and electron-deficient π-systems. Regarding the unanticipated finding of the binding energy order in X_3_–C–H⋯π-system complexes, NCI plots presented evidence of other noncovalent interactions between the three X atoms and the carbon ring of BZN, TFB and HFB. This noncovalent interaction was largest in the case X = I and lowest in the case X = Cl, while it was absent in the case X = F. Consequently, it resulted in the largest and lowest binding energy in I_3_–C–H⋯ and F_3_–C–H⋯π-system complexes, respectively (see Fig. 6).

For all halogen bond containing complexes, noncovalent interaction was observed between the halogen atom and the carbon ring. This is despite the various nucleophilic and electrophilic characters of the π-systems (see Fig. 6).

For F–T–F_3_⋯π-system complexes, the RDG isosurfaces were shaped as “fan-like” patterns. These patterns indicated interaction between the T atom and the π-system. Three additional disc-like isosurfaces emerged to indicate interaction between the coplanar F atoms in F–T–F_3_ monomer and the opposing carbon atoms of the π-system. This confirms the contribution of F_3_ atoms to F–T–F_3_⋯π-system binding energies.

### 3.5 Interplay of noncovalent interactions

**Fig. 7:**
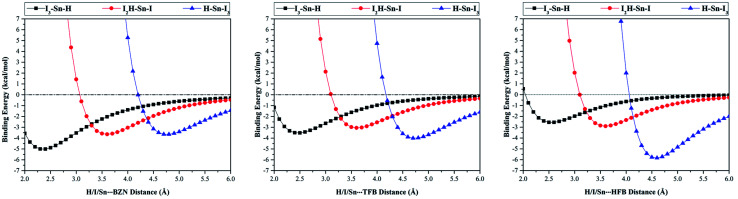
Binding energies calculated at MP2/aug-cc-pVDZ(PP) level of theory in kcal mol^−1^ for I_3_–Sn–H⋯, I_2_H–Sn–I⋯ and H–Sn–I_3_⋯π-system complexes (where π-system = BZN, TFB and HFB) at H/I/Sn⋯π-system distances from 2.0 to 6.0 Å with a step size of 0.1 Å.

In the context of the obtained results, a further study was appended to examine the interplay between hydrogen, halogen and tetrel interactions with π-systems. For comparable results, I_3_–Sn–H monomer was chosen to interact with BZN, TFB and HFB through the H, I and Sn atoms, forming hydrogen, halogen and tetrel bond interactions, respectively. A potential energy surface scan was performed in H/I/Sn⋯π-system bond in the range of 2.0 Å to 6.0 Å from the π-system centroid and with a step size of 0.1 Å. Results are depicted in Fig. 7 and binding energies at most favourable distances are tabulated in Table 4.

**Table 4:** Binding energies calculated at MP2/aug-cc-pVDZ(PP) level of theory for I_3_–Sn–H⋯, I_2_H–Sn–I⋯ and H–Sn–I_3_⋯π-system complexes at the most favorable H/σ-atom⋯π-system distance

Noncovalent interaction	π-system	Bond donors	Distance^a^ (Å)	*E*_Binding_ (kcal mol^−1^)
H/I/Sn⋯π-system	BZN	I_3_–Sn–H	2.40	−5.01
		I_2_H–Sn–I	3.60	−3.65
		H–Sn–I_3_	4.80	−3.65
	TFB	I_3_–Sn–H	2.50	−3.52
		I_2_H–Sn–I	3.60	−3.06
		H–Sn–I_3_	4.70	−4.01
	HFB	I_3_–Sn–H	2.60	−2.55
		I_2_H–Sn–I	3.60	−2.91
		H–Sn–I_3_	4.60	−5.84

a The most favorable at H/I/Sn⋯π-system distance based on the depicted curves in Fig. 7.

Considering binding energies with BZN, it was found that the I_3_–Sn–H⋯BZN interaction was strongest followed by the H–Sn–I_3_⋯BZN and the HI_2_–Sn–I⋯BZN with values −5.01, −3.65 and −3.65 kcal mol^−1^, respectively. In the case of π-system = HFB, binding energies of hydrogen and halogen bond donors with the π-system were reduced to −2.55 and −2.91 kcal mol^−1^ while the binding energy of the tetrel bond donor with HFB increased to −5.84 kcal mol^−1^. Generally, it was observed that, with the exception of the H–Sn–I_3_⋯π-system interactions, binding energy decreased as the π-system became more electron-deficient. This reversed trend of H–Sn–I_3_⋯π-system binding energies is interpretable in light of the large contribution of I_3_ interactions to the total binding energy. Based on chemical rationale, the interactions of I_3_ atoms with carbon atoms of the π-system are greater as more electron-withdrawing groups are attached to the carbons of the π-system.

From Table 4, the binding energies of I_3_–Sn–H/HI_2_–Sn–I/H–Sn–I_3_⋯TFB are in rather close proximity to each other. Binding energy had the order H–Sn–I_3_⋯TFB > I_3_–Sn–H⋯TFB > HI_2_–Sn–I⋯TFB with values of −4.01, −3.52 and −3.06 kcal mol^−1^, respectively. The mixed nucleophilic/electrophilic character of TFB, deduced previously from PoC results, may be the reason for this comparable outcome of binding energies.

## 4. Conclusions

In this study, interactions of hydrogen, halogen and tetrel bond donors with electron-rich and electron-deficient π-systems were investigated and compared. To assess the electrophilic and nucleophilic characters of the studied molecules, molecular electrostatic potential (MEP), maximum positive electrostatic potential (*V*_s,max_) and Point-of-Charge calculations were carried out. Moreover, potential energy surfaces for X_3_–C–H⋯, F_3_–C–X⋯ and F–T–F_3_⋯π-system complexes (where X = F, Cl, Br and I; T = C, Si, Ge and Sn; and π-system = benzene, 1,3,5-trifluorobenzene and hexafluorobenzene) were generated and the binding energies were calculated. The quantum theory of atoms in molecules (QTAIM) and the noncovalent interaction (NCI) index calculations were utilized to investigate the nature of the interactions. According to the results: (i) X_3_–C–H⋯π-system complexes showed unexpected binding energy pattern where binding energies increased (*i.e.*, more negative) with increase in X atomic size. This was explained by NCI analysis as the incorporation of halogen atoms in noncovalent interactions with the carbon ring of the π-systems; (ii) the binding energy of F_3_–C–X⋯ and F–T–F_3_⋯π-system increased as the σ-hole size of X and T atoms increased; (iii) the binding energies were, in general, larger for more electron-rich π-systems (*i.e.*, in the order BZN > TFB > HFB); (iv) binding energy calculations of F_3_–C–F⋯π-systems revealed the prevalence of the fluorine nucleophilic character; (v) for the tetrel bond-containing complexes, F–T–F_3_⋯π-system interactions could not be elucidated as σ-hole⋯π-system interaction only due to the participation of F_3_ atoms in interaction with the opposing carbon atoms of the π-system ring; (vi) QTAIM and NCI index supported the findings in a complementary way; and (vii) the hydrogen and halogen interaction strengths of I_3_–Sn–H bond donor with BZN, TFB and HFB correlated with the electron-richness of the π-system while the tetrel interaction strength of the same monomer correlated with the electron-deficiency of the π-system. These findings can be of advantage to more applied fields like materials science and drug discovery.

## Conflicts of interest

There are no conflicts to declare.

## Supplementary Material

RA-009-C9RA08007D-s001
