# Supplementary material for: Comparative investigation of interactions of hydrogen, halogen and tetrel bond donors with electron-rich and electron-deficient π-systems
Source: RSC Adv. 2019 Oct 15;9(56):32811–20. doi: 10.1039/c9ra08007d (PMC9073150; doi:10.1039/c9ra08007d)
Supplement: RA-009-C9RA08007D-s001 [file RA-009-C9RA08007D-s001.pdf]

Electronic Supplementary Material (ESI) for RSC Advances.
This journal is © The Royal Society of Chemistry 2019

Comparative investigation of interactions of hydrogen, halogen and tetrel bond donors with electron-rich and electron-deficient π-systems

Received 00th January 20xx,
Accepted 00th January 20xx

DOI: 10.1039/x0xx00000x

Mahmoud A. A. Ibrahim,*[a] Ossama A. M. Ahmed,[a,b] Nayra A. M. Moussa,[a] Sabry El-Taher[b] and Hussien Moustafa[b]

[a] Chemistry Department, Faculty of Science, Minia University, Minia 61519, Egypt.
[b] Department of Chemistry, Faculty of Science, Cairo University, Giza, Egypt.
† Electronic Supplementary Information (ESI) available: [details of any supplementary information available should be included here]. See DOI: 10.1039/x0xx00000x

Please do not adjust margins

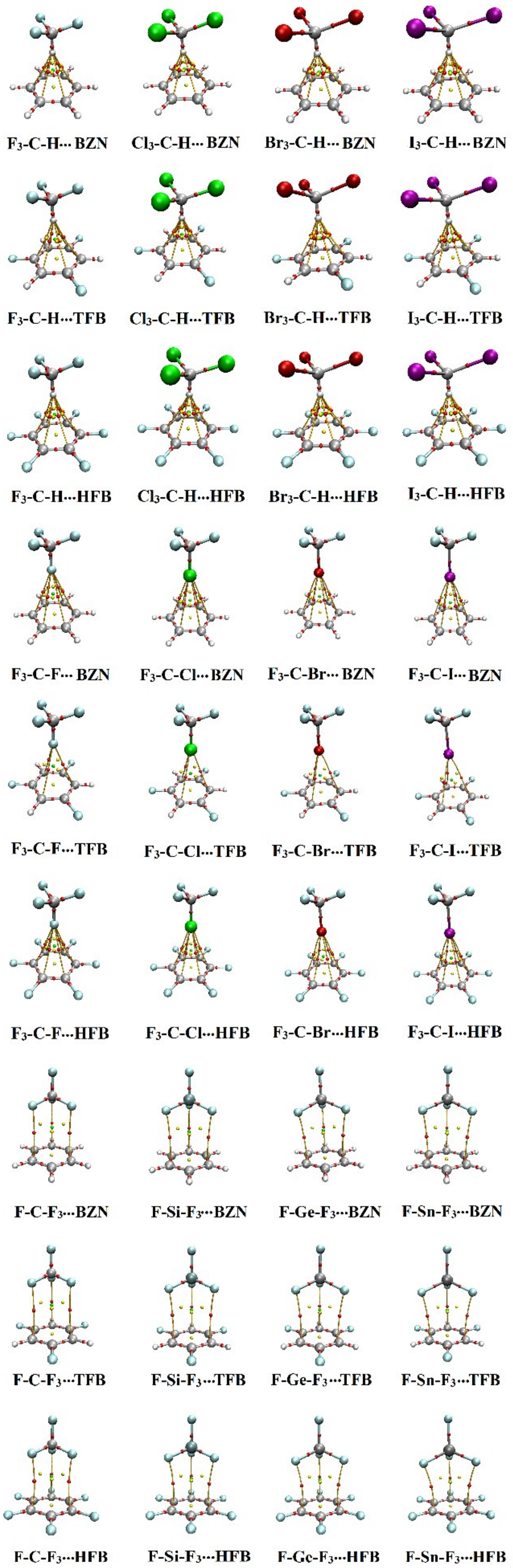

Fig. S1. QTAIM diagrams of X_3-C-H···, F_3-C-X··· and F-T-F_3···π-system (where X = F, Cl, Br and I, T = C, Si, Ge and Sn, and π-system = BNZ, TFB, and HFB) complexes. Red dots indicate the locations of bond critical points at the bond paths between the monomers at the most favorable H/σ-atom···π-system distance.

Please do not adjust margins

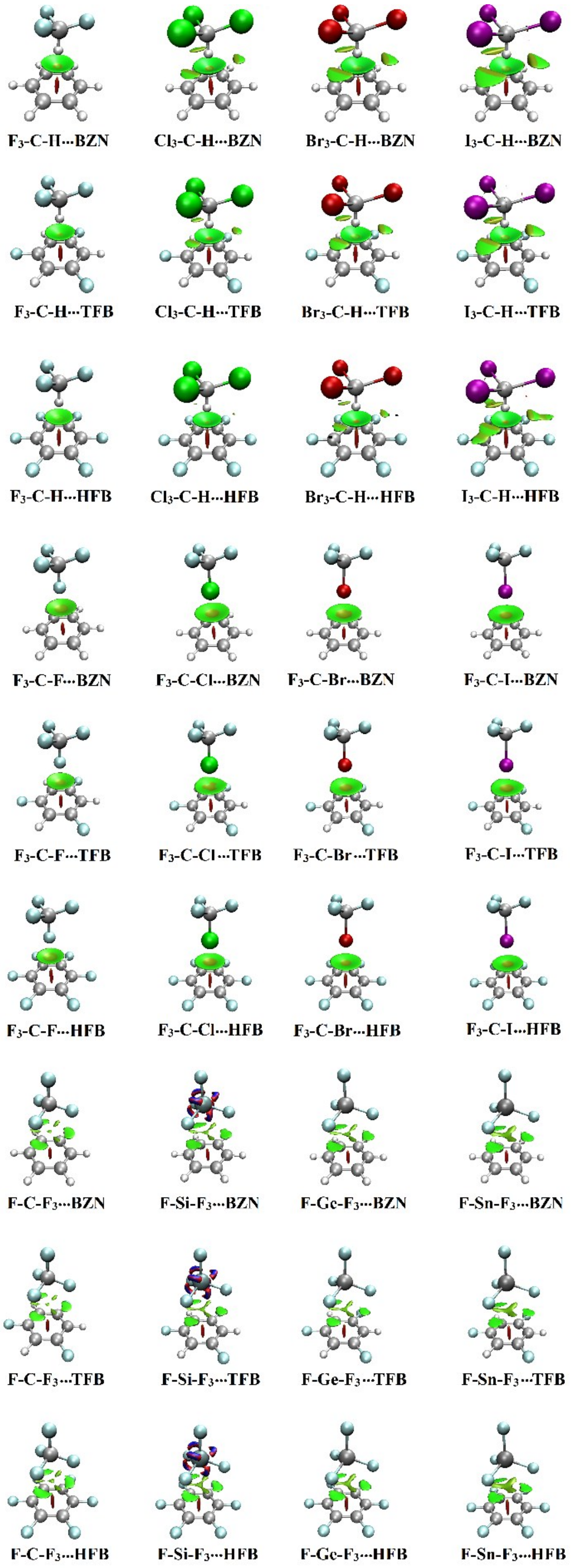

Fig. S2. Noncovalent interaction (NCI) diagrams of X_3-C-H···, F_3-C-X···, and F-T-F_3···π-system (where X = F, Cl, Br and I, T = C, Si, Ge and Sn, and π-system = BNZ, TFB, and HFB) complexes. The isosurfaces are plotted with a reduced density gradient value of 0.50 au and they are colored from blue to red according to sign(λ_2)ρ ranging from −0.035 to 0.020 au.
